# *RFC2* may contribute to the pathogenicity of Williams syndrome revealed in a zebrafish model

**DOI:** 10.1016/j.jgg.2024.09.016

**Published:** 2024-12

**Authors:** Ji-Won Park, Tae-Ik Choi, Tae-Yoon Kim, Yu-Ri Lee, Dilan Wellalage Don, Jaya K. George-Abraham, Laurie A. Robak, Cristina C. Trandafir, Pengfei Liu, Jill A. Rosenfeld, Tae Hyeong Kim, Florence Petit, Yoo-Mi Kim, Chong Kun Cheon, Yoonsung Lee, Cheol-Hee Kim

**Affiliations:** a Department of Biology, Chungnam National University, Daejeon 34134, Republic of Korea; b Department of Pediatrics, The University of Texas at Austin Dell Medical School, Austin, TX 78723, USA; c Department of Molecular and Human Genetics, Baylor College of Medicine, Houston, TX 77030, USA; d Baylor Genetics Laboratories, Houston, TX 77021, USA; e Department of Pediatrics, Kyung Hee University Hospital at Gangdong, Seoul 05278, Republic of Korea; f Univ. Lille, CHU Lille, Clinique de génétique Guy Fontaine, F-59000 Lille, France; g Department of Pediatrics, Chungnam National University Sejong Hospital, Sejong 30099, Republic of Korea; h Department of Pediatrics, Pusan National University Children's Hospital, Yangsan 50612, Republic of Korea; i Research Institute for Convergence of Biomedical Science and Technology, Pusan National University Yangsan Hospital, Yangsan 50612, Republic of Korea; j Clinical Research Institute, Kyung Hee University Hospital at Gangdong, School of Medicine, Kyung Hee University, Seoul 05278, Republic of Korea

**Keywords:** Williams syndrome, RFC2, RFC5, Zebrafish, Knockout, CRISPR-Cas9

## Abstract

Williams syndrome (WS) is a rare multisystemic disorder caused by recurrent microdeletions on 7q11.23, characterized by intellectual disability, distinctive craniofacial and dental features, and cardiovascular problems. Previous studies have explored the roles of individual genes within these microdeletions in contributing to WS phenotypes. Here, we report five patients with WS with 1.4 Mb–1.5 Mb microdeletions that include *RFC2*, as well as one patient with a 167-kb microdeletion involving *RFC2* and six patients with intragenic variants within *RFC2*. To investigate the potential involvement of *RFC2* in WS pathogenicity, we generate a *rfc2* knockout (KO) zebrafish using CRISPR-Cas9 technology. Additionally, we generate a KO zebrafish of its paralog gene, *rfc5*, to better understand the functions of these *RFC* genes in development and disease. Both *rfc2* and *rfc5* KO zebrafish exhibit similar phenotypes reminiscent of WS, including small head and brain, jaw and dental defects, and vascular problems. RNA-seq analysis reveals that genes associated with neural cell survival and differentiation are specifically affected in *rfc2* KO zebrafish. In addition, heterozygous *rfc2* KO adult zebrafish demonstrate an anxiety-like behavior with increased social cohesion. These results suggest that *RFC2* may contribute to the pathogenicity of WS, as evidenced by the zebrafish model.

## Introduction

Williams syndrome (WS), also known as Williams-Beuren syndrome (WBS; OMIM: 194050), is one of the common microdeletion syndromes occurring in approximately 1 in 7500 individuals worldwide (Morris et al., 2020). WS is caused by a recurrent microdeletion of 1.55 Mb–1.8 Mb on chromosome 7q11.23, affecting around about 28 genes, of which the roles of only a few genes have been elucidated (Morris et al., 2020; Kozel et al., 2021). This multi-systemic disorder is characterized by typical facial features, including bitemporal narrowing, broad forehead, epicanthal folds, periorbital swelling, puffy cheeks, short nose, prominent nasal tip, long philtrum, wide mouth, widely spaced teeth, and small chin. Patients often have cardiovascular issues, including supravalvular aortic stenosis, hypertension, and peripheral pulmonary or other artery stenosis, as well as connective tissue abnormalities like hoarse voice, umbilical or inguinal hernia, joint laxity or limitation, and bladder diverticula. Furthermore, endocrine dysfunctions include hypercalcemia, hypothyroidism, early puberty, growth deficiency, and diabetes mellitus; auditory problems range from hypersensitivity to sound to sensorineural hearing loss to recurrent otitis media. Intellectual disability is a key feature, often accompanied by behavioral traits such as overfriendliness, anxiety, and attention-deficit/hyperactivity disorder are also common (Morris et al., 2020).

Over the past few decades, research has focused on understanding the roles that individual genes within the copy-number variation (CNV) region might be playing in the WS phenotypes. For instance, *BAZ1B* has been studied in both zebrafish and mice in relation to WS. Zebrafish with *baz1b* loss of function showed developmental defects with craniofacial features along with altered social behaviors (Torres-Pérez et al., 2023). Similarly, *Baz1b* deficienc*y* in mice resulted in growth retardation, agnathia and ocular defects, and dilated bowel (McIntosh et al., 2022; Pai et al., 2024). Studies on *DNAJC30* showed that the removal of *Dnajc30* in mice resulted in hypofunctional mitochondria, diminished morphological features of neocortical pyramidal neurons, and altered behaviors reminiscent of WS (Tebbenkamp et al., 2018). *ELN*, encoding elastin, an essential structure for elastic fibers in the connective tissue, is a causative gene for cardiovascular and connective tissue symptoms in WS (Ewart et al., 1993; Duque Lasio and Kozel, 2018). Additionally, *LIMK1*, encoding LIM domain kinase 1, is linked to poor visuospatial cognitive deficits observed in WS (Frangiskakis et al., 1996; Gregory et al., 2019). Research on *EIF4H*, encoding a protein related to protein synthesis, suggests its contribution to certain deficits associated with WS. *Eif4h* knockout (KO) mice displayed growth retardation, microcephaly, and severe impairments of fear-related associative learning and memory formation (Capossela et al., 2012). *CLIP2*, along with the transcription factor genes *GTF2I* and *GTF2IRD1*, has been implicated in the visuospatial and cognitive aspects of WS (Vandeweyer et al., 2012). These transcription factor genes have also been associated with intellectual ability, social functioning, and anxiety in WS (Heller et al., 2003; Antonell et al., 2010; Sakurai et al., 2011; Kozel et al., 2021).

Replication Factor C (RFC) is a pentameric protein complex essential for DNA replication, acting as a clamp loader that facilitates the attachment of proliferating cell nuclear antigen (PCNA) onto DNA, a critical step for subsequent DNA synthesis (Mossi et al., 1997; Yao et al., 2006). RFC consists of one large subunit (RFC1) and four smaller subunits (RFC2, RFC3, RFC4, and RFC5), which are homologous to each other and belong to the AAA^+^ family of ATPases (Neuwald et al., 1999). A repeat expansion in *RFC1* causes the autosomal recessive cerebellar ataxia with neuropathy and vestibular areflexia syndrome (CANVAS), a slowly progressive multisystem neurodegenerative disorder (Dupre et al., 2021; Delforge et al., 2024). Recently, the association between one of the small subunits of the RFC complex (RFC2–RFC5) and disease has been reported. Bi-allelic loss-of-function variants in the *RFC4* gene were discovered in individuals with incoordination and muscle weakness, hearing impairment, and decreased body weight (Morimoto et al., 2024).

Zebrafish (*Danio rerio*) is a powerful model organism for the study of genes involved in neuropsychiatric disorders (Kalueff et al., 2014; Choi et al., 2021). They are a well-characterized model for studying autism spectrum disorders (ASD) (Kim et al., 2017; Choi et al., 2018) and to validate the function of human candidate genes implicated in developmental disorders (May et al., 2015; Lee et al., 2021). Moreover, major human brain regions are anatomically and functionally conserved in fish, including the amygdala, habenula, and cerebellum (Bae et al., 2009; Geng and Peterson, 2019). Recently, we used zebrafish KO model to functionally validate *FAM50A*, the causative gene for the Armfield X-linked intellectual disability (XLID) syndrome (Lee et al., 2020). The XLID syndrome is characterized by distinctive phenotypes involving multiple systems: growth retardation, prominent forehead and dysmorphic facial features, and ocular abnormalities and seizures. Through pull-down assay and mass spectrometry analysis using FAM50A as bait, we identified RFC3 and RFC5 as components of the FAM50A complex, suggesting potential roles for *RFC* genes in developmental disorders, including intellectual disability (Lee et al., 2020). Interestingly, a comprehensive literature review revealed that *RFC2*, a paralog of *RFC5*, is localized within the WS critical region (WSCR) on 7q11.23. Here, we report five patients with WSCR microdeletions, one patient with a smaller deletion comprising *RFC2*, and six cases featuring intragenic variants in *RFC2*. To investigate a possible role of *RFC2* in WS pathogenicity and developmental disorders, we generated a *rfc2* KO zebrafish using CRISPR-Cas9 technology. To further explore the importance of these *RFC* genes in developmental processes and diseases, we also generated *rfc5* KO zebrafish.

## Results

### Five WS patients with the WSCR microdeletions

**Fig. 1:**
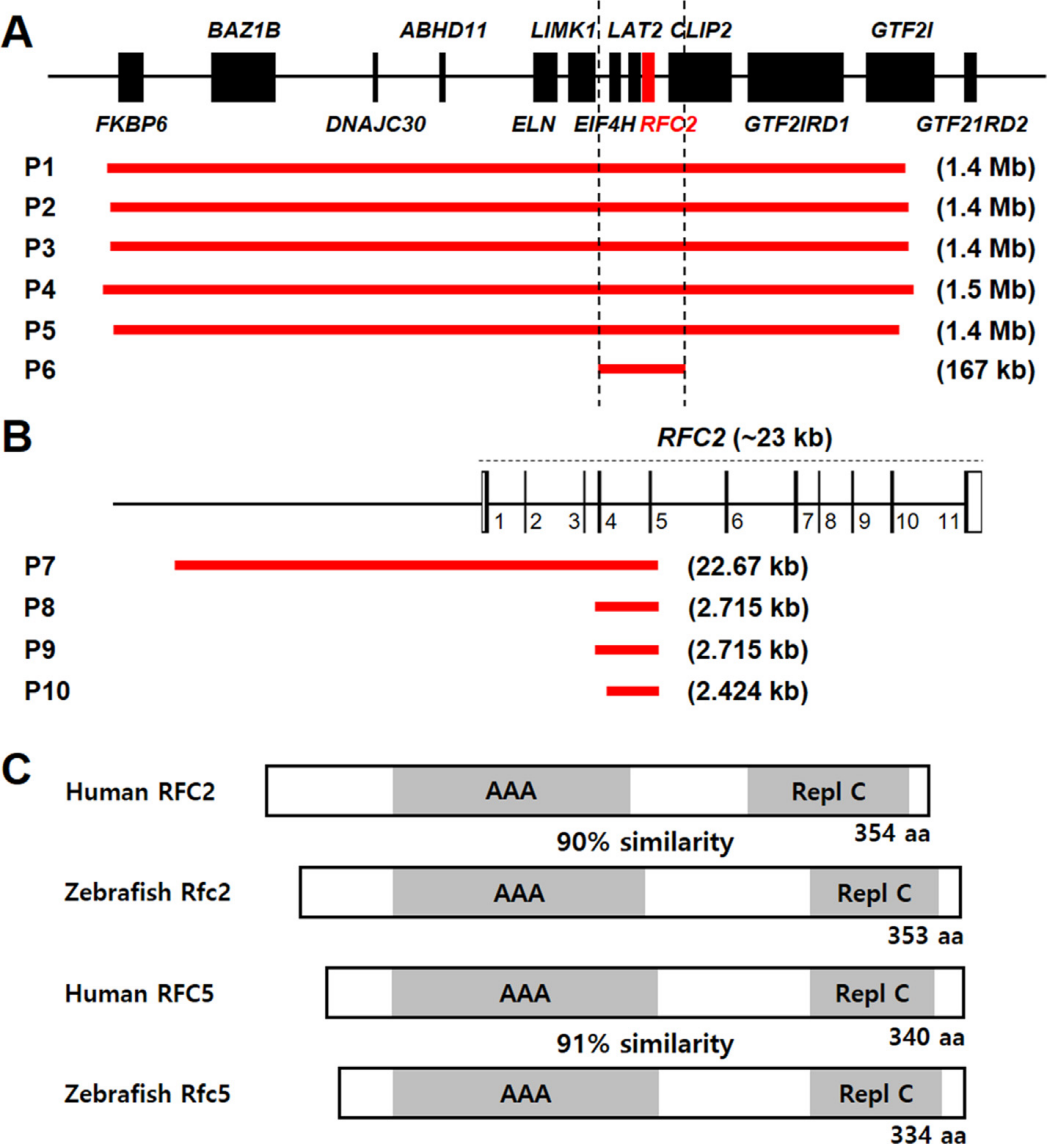
WS patients with 7q11.23 microdeletions and *RFC2* intragenic deletions. **A**: Five WS patients (P1–P5) with typical 7q11.23 microdeletions. Chromosomal microarray test detected 1.4 Mb–1.5 Mb microdeletions including *RFC2*. We also identified one patient (P6) with a smaller 167 kb deletion, comprising *RFC2* but not *ELN*. **B**: Patients (P7–P10) with *RFC2* intragenic microdeletions. *RFC2* consists of 11 exons (1–11), spanning ∼23 kb. **C**: Comparison of human RFC2 (NP_852136.1) with zebrafish Rfc2 (NP_001013344.2) and human RFC5 (NP_031396.1) with zebrafish Rfc5 (NP_001003862.1). All four have an AAA domain and a Repl C domain in common. The zebrafish *rfc2* encodes 353-aa protein, with a high level of similarity (90%) to the human RFC2 protein and the zebrafish *rfc5* encodes 334-aa protein, with a high level of similarity (91%) to the human RFC5 protein. aa, amino-acid; Repl C, Replication factor C C-terminal domain; AAA, AAA^+^ ATPase domain.

We have identified five patients (P1–P5) with the typical 1.4 Mb–1.5 Mb microdeletion in the WSCR, which includes the *RFC2* gene. These patients displayed characteristic WS phenotypes with facial features of a broad forehead, underdeveloped chin, short nose, and full cheeks. Patients had microcephaly or encephalopathy, dental issues, connective tissue abnormalities, diverse arteriopathy, developmental delay, and speech delay (Fig. 1A).

**Patient 1)** He was a 32-month-old male who was born at 38 + 6 weeks, weighing 3.14 kg. He presented with growth failure. Echocardiography showed supravalvular aortic stenosis and peripheral pulmonary artery stenosis. He had a typical WS facial appearance, microcephaly (46 cm, <3rd percentile), a small jaw, and small teeth. He showed connective tissue abnormalities, including joint laxity. He is receiving therapies for developmental delay. He was diagnosed with WS by chromosomal microarray (CytoScan Dx Assays), which showed a 7q11.23 microdeletion including *RFC2* (arr 7q11.23[72,700,524–74,142,190] x 1 [1.4 Mb deletion], reference genome GRCh37/hg19).

**Patient 2)** She was a 51-month-old female who was born at 37 + 4 weeks, weighing 2.15 kg. She presented with anorectal malformation with a perianal fistula. Echocardiography showed supravalvular aortic stenosis and peripheral pulmonary artery stenosis. She had a typical WS facial appearance, microcephaly (47 cm, <3rd percentile), a small jaw, and small teeth. She had bilateral renal hypoplasia and connective tissue abnormalities, including joint laxity. She has been receiving therapies for developmental delay. She was diagnosed with WS by chromosomal microarray (CytoScan Dx Assays), which showed a 7q11.23 microdeletion including *RFC2* (arr 7q11.23[72,701,084–74,142,190] x 1 [1.4 Mb deletion], reference genome GRCh37/hg19).

**Patient 3)** He was a 28-year-old male who was born at 39 weeks, weighing 3.21 kg. He presented with heart failure. Echocardiography showed severe mitral valve prolapse and dilated cardiomyopathy. He had a typical WS facial appearance, microcephaly (52 cm, <3rd percentile), small jaw, clinodactyly, and severe short stature (153 cm, <3rd percentile). He had connective tissue abnormalities, including joint laxity. He also had skeletal abnormalities such as scoliosis and varus alignment of the knee. He was diagnosed with WS by chromosomal microarray (CytoScan Dx Assays), which showed a 7q11.23 microdeletion including *RFC2* (arr 7q11.23[72,701,018–74,143,240] x 1 [1.4 Mb deletion], reference genome GRCh37/hg19).

**Patient 4)** He was a 3-year-old male who was born at 37 + 2 weeks, weighing 2.67 kg, and presented with cyanosis at birth. Echocardiography showed supravalvular aortic stenosis and bilateral peripheral pulmonary artery stenosis. He had a typical WS facial appearance, microcephaly (36.5 cm, <3rd percentile), small chin, and short stature. He has been receiving therapies for developmental delay. He was diagnosed with WS by chromosomal microarray (CytoScan Dx Assays), which showed a 7q11.23 microdeletion including *RFC2* (arr 7q11.23[72,643,519–74,154,209] x 1 [1.5 Mb deletion], reference genome GRCh37/hg19). At 3 years of age, he had short stature (88.9 cm, <3rd percentile), low body weight (10.1 kg, <3rd percentile), and global developmental delay.

**Patient 5)** She was a 5-year-old female who was born at 40 + 1 weeks of gestational age, weighing 2.8 kg and presenting with a heart murmur and polydactyly of the right thumb at birth. Echocardiography showed supravalvular aortic stenosis and peripheral pulmonary artery stenosis. She had a typical WS facial appearance, small chin, and microcephaly (34 cm, <3rd percentile). She was diagnosed with WS by chromosomal microarray (CytoScan Dx Assays), which showed a 7q11.23 microdeletion including *RFC2* (arr 7q11.23[72,725,001–74,125,000] x 1 [1.4 Mb deletion], reference genome GRCh37/hg19). After diagnosis, global developmental delay was found during follow-up, and she is receiving therapies. At 5 years of age, she had global developmental delay, multiple dental caries, and growth retardation (height 96.9 cm, <1st percentile; weight 13.5 kg, <3rd percentile).

### One patient with a 167-kb deletion within the WSCR

**Patient 6)** He was a 4.5-year-old male with non-syndromic intellectual disability. He was born at 38 weeks of gestational age, with a birth weight of 2.95 kg, length of 47 cm, and Occipital Frontal Circumference (OFC) of 35.5 cm. Hypotonia was diagnosed at three months leading to physiotherapy. He had global developmental delay, with walking achieved at 18.5 months and first words emerging around 3 years of age. At 4.5 years, he only had non-specific use of a few words and had global and fine motor difficulties with pes planus and frequent falls. He had behavioral troubles like aggression, attention deficit, and paroxysmal laughter. His growth was normal, with height at +1.5 SD, weight at +1.5 Standard Deviation (SD), and OFC at +2 SD. He had no eye problem, and the eye examination was normal. He had no heart defect and no facial dysmorphism. The patient had a 167.21 kb microdeletion on chromosome 7 (73,579,058–73,746,262, reference genome GRCh37/hg19), including *RFC2*, but not comprising the *ELN* gene. This deletion was inherited from the mother who was also affected by intellectual disability. Patient information with accession number 436716 was deposited in the DECIPHER database (https://www.deciphergenomics.org/).

### Intragenic variants in the *RFC2* gene

Chromosomal microarray analysis (CMA) is used for the detection of clinically significant microdeletions or duplications, with a high sensitivity for submicroscopic aberrations. CMA is increasingly used for uncovering copy number variants thought to play an important role in the pathogenesis of a variety of disorders, primarily developmental delay/intellectual disability, ASD, and multiple congenital anomalies. Through a search of results from clinical CMA testing and clinical whole-exome sequencing, we identified six patients having intragenic variants, four microdeletions (Fig. 1B), and two stop-gain variants within *RFC2*.

**Patient 7)** He was a 6-month-old male with autosomal anomalies. Clinical CMA (Oligo V8.1.1) identified a minimum size of 22.67 kb (73,660,767–73,683,437; maximum, 73,657,895–73,731,391, reference genome GRCh37/hg19) microdeletion on chromosome 7, spanning exons 1–5 of *RFC2*. No other clinically significant CNVs were identified.

**Patient 8)** He was a 5-year-old male with developmental delay, ASD, and hypopigmentation of hair. Clinical CMA (Oligo V8.1) identified a minimum size of 2.715 kb (73,660,766–73,663,481; maximum, 73,657,896–73,664,146, reference genome GRCh37/hg19) microdeletion on chromosome 7, spanning exon 4 and exon 5 of *RFC2*. No other clinically significant CNVs were identified. Maternally inherited.

**Patient 9)** He was a 2-year-old male with developmental delay, speech delay, micrognathia, and a family history of chromosomal abnormality. Clinical CMA (Oligo V8.1) identified a minimum size of 2.715 kb (73,660,766–73,663,481; maximum, 73,657,896–73,664,146, reference genome GRCh37/hg19) microdeletion on chromosome 7, spanning exon 4 and exon 5 of *RFC2*. No other clinically significant CNVs were identified. Paternally inherited.

**Patient 10)** He was a 10-year-old male with autism. CMA high-resolution (HR) + single nucleotide polymorphism (SNP) screen (CMA-HR + SNP, V9.1.1) identified a minimum size of 2.424 kb (73,660,767–73,663,191; maximum, 73,657,844–73,663,303, reference genome GRCh37/hg19) microdeletion on chromosome 7, spanning exon 5 of *RFC2*. No other clinically significant CNVs were identified.

**Patient 11)** He was a 10-year-old male with joint hypermobility, easy bruising, postural orthostatic tachycardia syndrome (POTS), dysautonomia, high arched palate, pes planus, translucent skin, gastrointestinal (GI) issues, abdominal pain and migraines, recurrent vomiting, and subsequent dehydration. Clinical whole-exome sequencing identified a point mutation in *RFC2*, c.919C>T (NM_181471); p.Q307X. It has not been Sanger confirmed, and parents have not been tested, but it was seen in 39/77 reads from the locus in the proband.

**Patient 12)** She was a 4-year-old female with developmental regression, febrile and afebrile generalized and multifocal tonic seizures, delayed motor milestones, delayed speech, truncal ataxia, and anemia. Brain MRI showed cerebellar malformation. Clinical whole-exome sequencing identified a point mutation in *RFC2*, c.910A>T (NM_181471); p.K304X. This variant has not been Sanger confirmed, and parents have not been tested, but it was seen in 31/59 reads from the locus in the proband.

### Generation of *rfc2* KO and *rfc5* KO zebrafish using CRISPR-Cas9

**Fig. 2:**
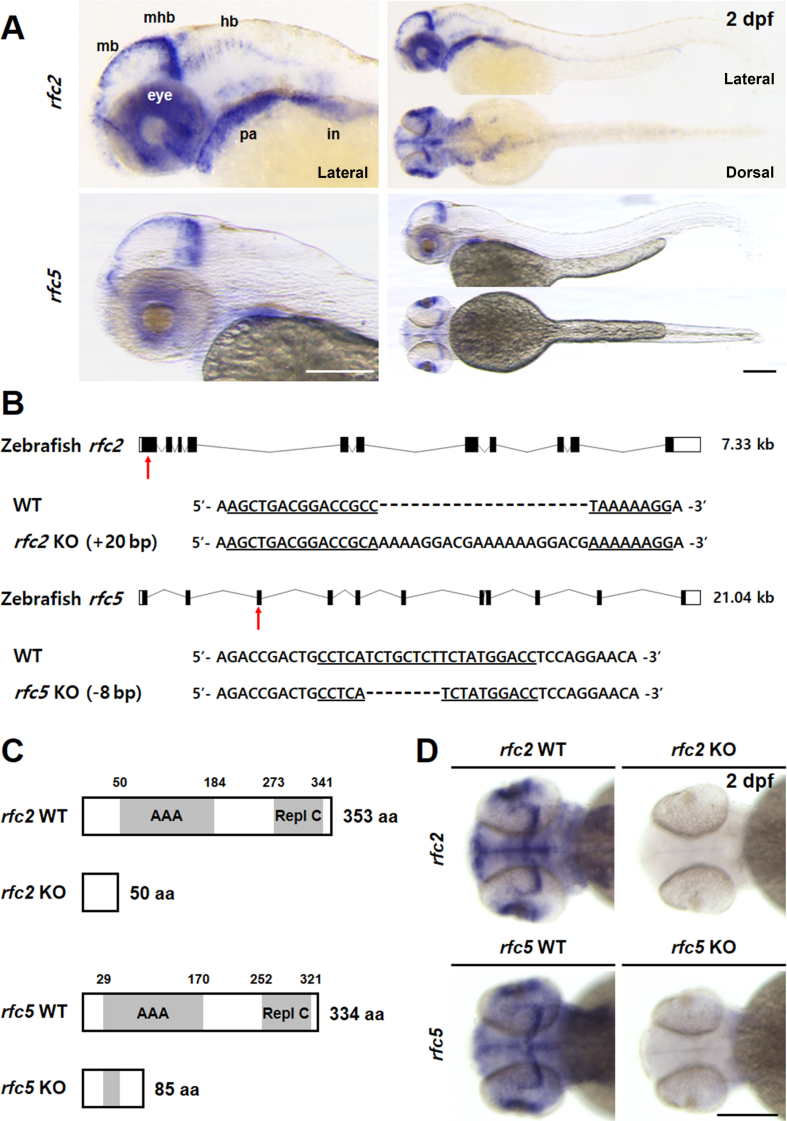
Generation of *rfc2* KO and *rfc5* KO zebrafish using CRISPR-Cas9. **A**: Expression of *rfc2* or *rfc5* mRNAs in zebrafish embryos at 2 dpf. Lateral and dorsal view, anterior is to the left. Both genes are expressed in mb, mhb, hb, and eyes. **B**: Schematic gene structure of zebrafish *rfc2* and *rfc5* with KO target sites (arrows), exon 1 of *rfc2* and exon 3 of *rfc5*. Indel mutations include 20-bp insertion (+22 bp, −2 bp) in *rfc2* (named *rfc2*^ck179a^) and 8-bp deletion in *rfc5* (named *rfc5*^ck171a^). **C**: Predicted protein structure of each KO mutation, resulting in truncated proteins caused by frameshift. **D**: Loss of *rfc2* or *rfc5* mRNA expression in each KO zebrafish at 2 dpf. Dorsal view. *n* = 12 for *rfc2* WT, *n* = 17 for *rfc2* KO, *n* = 12 for *rfc5* WT, and *n* = 9 for *rfc5* KO. Scale bar, 200 μm (**A** and **D**). mb, midbrain; mhb, midbrain-hindbrain boundary; hb, hindbrain; WT, wild-type; KO, knockout; dpf, days post fertilization; AAA, AAA^+^ ATPase domain; Repl C, Replication factor C C-terminal domain; pa, pharyngeal arches; in, intestine.

To validate the biological function of *RFC* genes in developmental disorders, we utilized a zebrafish animal model. Zebrafish comprises orthologs of human *RFC2* and *RFC5*, which encode proteins of 353 and 334 amino acids in zebrafish, respectively. Zebrafish Rfc proteins have a very high homology to their human counterparts, showing more than 90% similarity (Fig. 1C). To characterize the spatiotemporal mRNA expression pattern of *rfc2* and *rfc5* genes during zebrafish development, we performed whole-mount in situ hybridization (WISH). We observed expression of *rfc2* restricted to the central nervous system (CNS) and other tissues from 1 day post fertilization (dpf). At 2 dpf, *rfc2* expression was detected mainly in the eyes, midbrain, midbrain-hindbrain boundary (MHB), hindbrain, pharyngeal arches, and intestine. The expression pattern of *rfc5* was similar to that of *rfc2* (Fig. 2A). The specific expression of *rfc2* and *rfc5* genes during early development suggested that their important roles in CNS development.

To evaluate the roles of *rfc2* and *rfc5*, we generated KO lines using the CRISPR-Cas9 system. We specifically disrupted the highly conserved AAA + ATPase domain by injecting Cas9 mRNA together with single guide RNAs aimed at exon 1 of *rfc2* and exon 3 of *rfc5*. After KO mutant screen, we finally established several KO alleles for each gene: 20-bp insertion (+22 bp, −2 bp), 73-bp deletion, and 14-bp deletion for *rfc2* (*rfc2* ^ck179a^, *rfc2* ^ck179b^, and *rfc2* ^ck179c^); 8-bp deletion and 10-bp insertion (+11 bp, −1 bp) for *rfc5* (*rfc5* ^ck171a^ and *rfc5* ^ck171b^). We used *rfc2* ^ck179a^ and *rfc5* ^ck171a^ for all subsequent experiments, referred to hereafter as *rfc2* KO and *rfc5* KO, respectively (Fig. 2B). These gene disruptions lead to frameshift mutations producing truncated proteins that lack the AAA + ATPase domain (Fig. 2C). WISH results confirmed that both *rfc2* and *rfc5* mRNA levels were undetectable in each KO zebrafish, likely due to nonsense-mediated mRNA decay (NMD) (Fig. 2D).

### Craniofacial phenotypes in *rfc2* KO and *rfc5* KO zebrafish

**Fig. 3:**
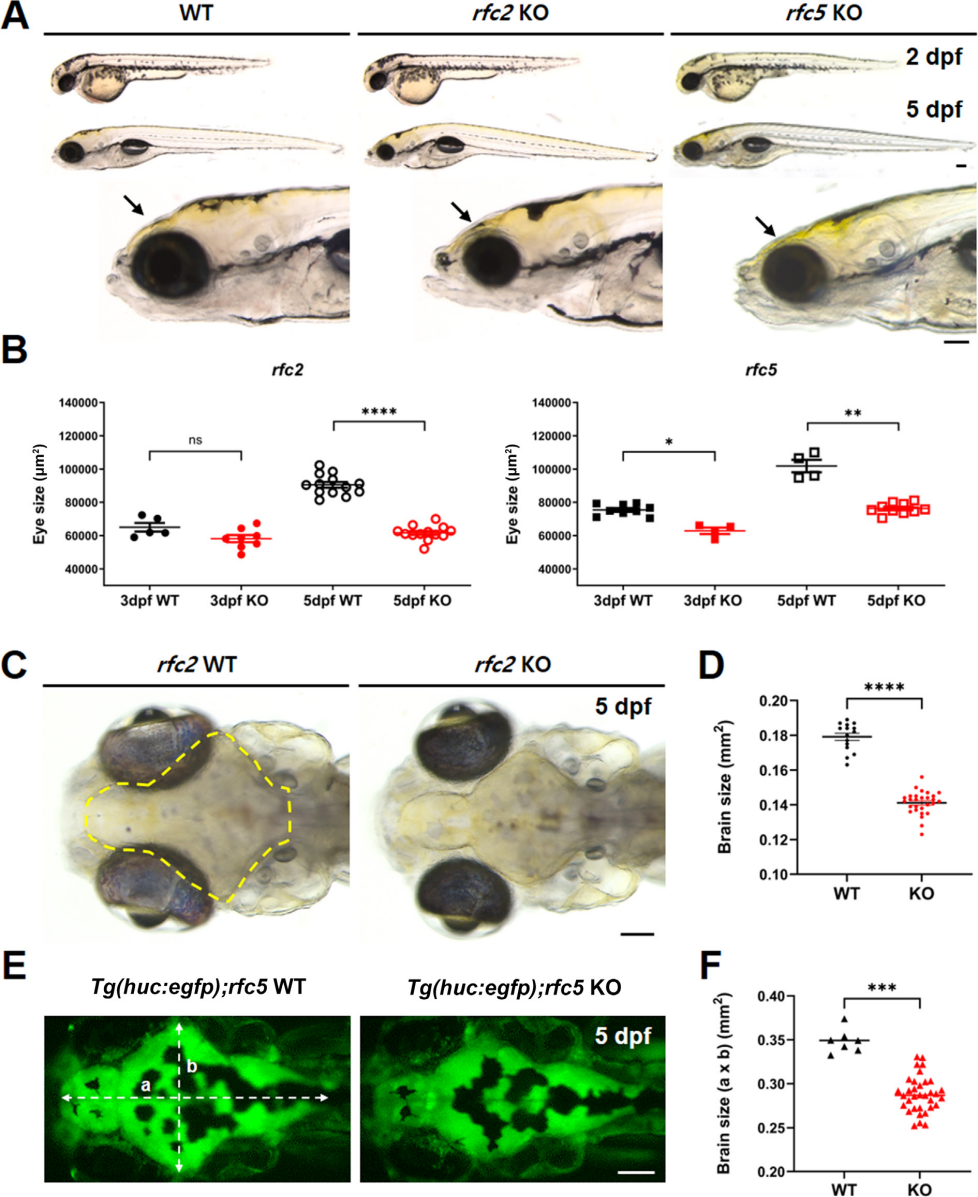
Reduced head and brain size in *rfc2* KO and *rfc5* KO zebrafish. **A**: Reduced head size in KO zebrafish is prominent at 5 dpf, whereas relatively comparable at 2 dpf. Eye size is also reduced in both KO zebrafish, compared to WT zebrafish at 5 dpf. *n* = 5 for *rfc2* KO and *n* = 4 for *rfc5* KO at 2 dpf. *n* = 13 for *rfc2* KO and *n* = 25 for *rfc5* KO at 5 dpf. Lateral view. **B**: Quantification of eye size in *rfc2* KO and *rfc5* KO zebrafish. The planar area of eye was measured by Image J software at 3 dpf and 5 dpf, respectively. **C**: Reduced brain size in *rfc2* KO zebrafish at 5 dpf. Dorsal view. **D**: Quantification of reduced brain size (bordered area in **C**) in *rfc2* KO zebrafish at 5 dpf. *n* = 18 for WT and *n* = 31 for KO. **E**: Representative fluorescent image of *rfc5* KO zebrafish crossed with neuron-specific GFP transgenic line *Tg(huc:egfp)* at 5 dpf. Dorsal view, anterior is to the left. **F**: Quantification of brain size in *rfc5* KO at 5 dpf. Relative brain size was obtained by multiplying the length of (a) and (b) marked in the brain image of (**E**). *n* = 7 for WT and *n* = 34 for KO. Data are presented as mean ± SEM. Statistical significance was determined by the Kruskal–Wallis tests with post-hoc Dunn's multiple comparisons tests. ns, not significant, *P* > 0.05; ∗, *P* < 0.05; ∗∗, *P* < 0.01; ∗∗∗, *P* < 0.001; ∗∗∗∗, *P* < 0.0001. Scale bar, 100 μm (**A**, **C**, **E**). dpf, days post fertilization; WT, wild-type; KO, knockout; SEM, standard error of the mean.

One of the most common characteristics of WS patients is their distinct craniofacial features, which include a broad forehead, puffiness around the eyes, a flat nasal bridge, full cheeks, and a small chin (Morris et al., 2020; Kozel et al., 2021). To determine whether the facial features of KO zebrafish resemble those of WS patients, we examined the early morphology of *rfc2* KO and *rfc5* KO zebrafish from 1 dpf to 5 dpf. Up to 2 dpf, the external gross morphology of both *rfc2* KO and *rfc5* KO was similar to that of their WT siblings. However, at 5 dpf, both KOs showed a significant reduction in head region, which resembles the retrusive midface in WS patients (Fig. 3A). Also, we observed smaller eyes in KO zebrafish (Fig. 3A and 3B), although ear size, swim bladder inflation, and trunk region were relatively normal, compared to WT siblings. We confirmed similar phenotypes in different mutant alleles by complementation tests both in *rfc2* KO and *rfc5* KO zebrafish (Fig. S1). Reduced head and brain size are common features seen in patients with WS (Jackowski et al., 2009), and similar reductions were observed in both KO models. To compare brain sizes, we utilized the transparency of larval zebrafish at 5 dpf, which enables clear imaging of internal organs (Veldman and Lin, 2008). Brain areas were quantified with the ImageJ software, and results indicated that *rfc2* KO zebrafish had significantly smaller brains compared to WT siblings (Fig. 3C and 3D). For further clarification, we used KO zebrafish crossed with a CNS-specific GFP transgenic line *Tg(huc:egfp)*, which exhibits green fluorescence in neuronal cells (Park et al., 2000). The brain size of *rfc5* KO zebrafish was significantly smaller compared to WT siblings (Fig. 3E and 3F). These results indicated that either *rfc2* or *rfc5* deficiency may be involved in neurodevelopment and related neurological diseases.

### Increased cell death and reduced neural progenitors in KO zebrafish

**Fig. 4:**
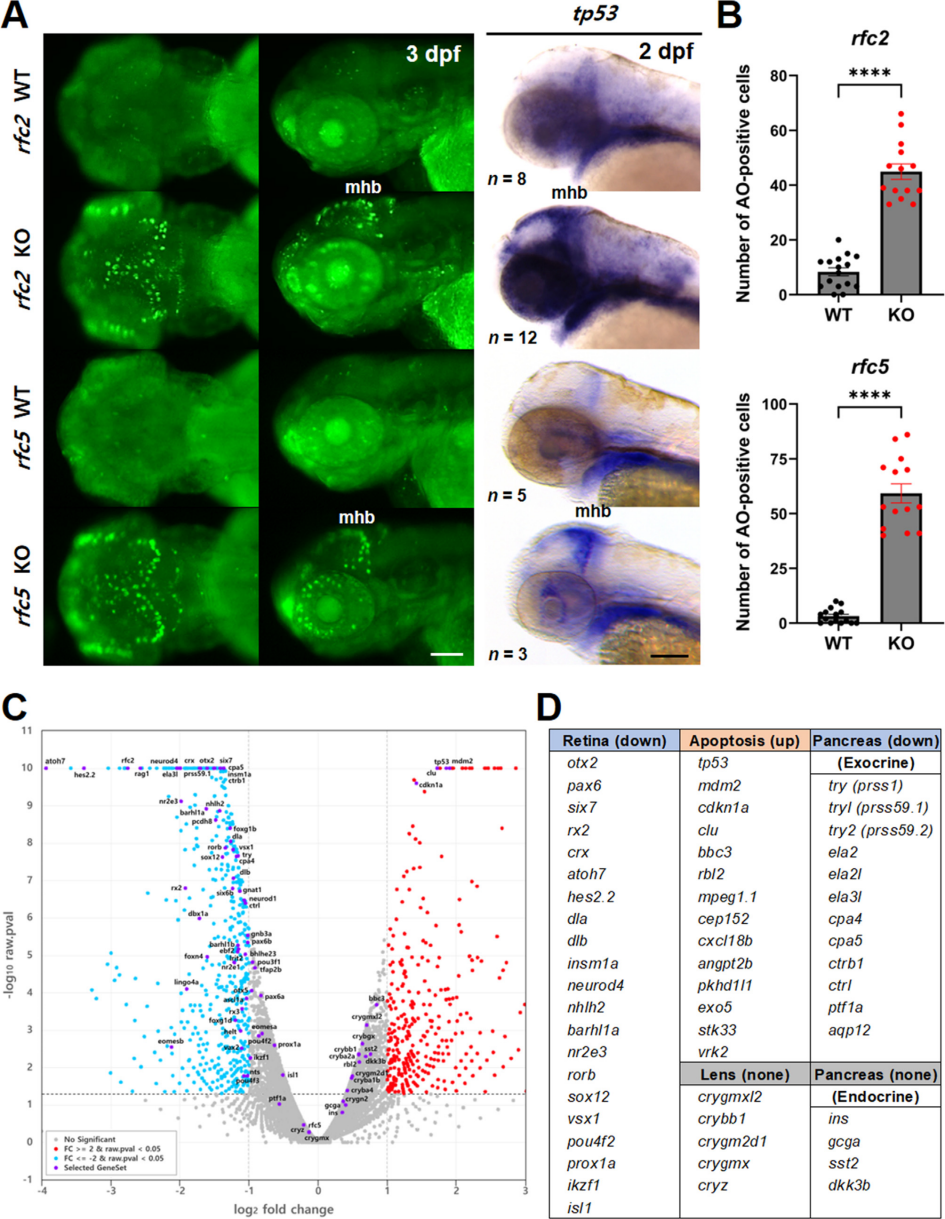
Increased cell death in *rfc2* KO and *rfc5* KO zebrafish. **A**: Detection of cell death by AO staining in KO zebrafish at 3 dpf. Apoptotic cells were mainly detected in midbrain, mhb, and retina. Earlier apoptotic cells were confirmed by the apoptosis marker *tp53* expression at 2 dpf. Left panels are dorsal view, middle and right panels are lateral view, anterior is to the left. **B**: Quantification of AO-positive cells within the brain region in *rfc2* KO or *rfc5* KO zebrafish. *n* = 16 for *rfc2* WT, *n* = 14 for *rfc2* KO, *n* = 15 for *rfc5* WT, and *n* = 14 for *rfc5* KO. Data are presented as mean ± SEM. Statistical significance was determined by the unpaired two-tailed *t*-tests with Welch's correction. ∗∗∗∗, *P* < 0.0001. **C**: Visualization of RNA-seq analysis with volcano plot. Blue dots represent down-regulated, red dots for up-regulated, gray dots for not significantly changed, and purple dots for labeled genes in *rfc2* KO, compared to WT. **D**: List of representative gene sets involved in eye development or pancreas function. Scale bar, 100 μm (**A**). AO, acridine orange; mhb, midbrain-hindbrain boundary; WT, wild-type; KO, knockout; SEM, standard error of the mean.

RFC2 and RFC5 are components of the replication factor C complex, crucial for DNA replication and repair, cell proliferation, regulation of cell cycle checkpoints, and cell growth under stress (Kelman and Kelman, 2013). Since both genes are expressed prominently in the MHB, a site of active cell proliferation in the brain (Gutzman et al., 2008), we investigated whether cell death or apoptosis contributes to the brain size reduction observed in the KO zebrafish. Apoptotic cells were detected in live zebrafish at 3 dpf using acridine orange (AO) staining with vivid fluorescent signals. Interestingly, apoptotic cells localized to the same areas as *rfc2* and *rfc5* expression, specifically in MHB and eye/retina (Figs. 2A and 4A). To confirm these AO-positive apoptotic cells, we examined the expression of an apoptotic molecular marker, *tp53*, encoding tumor protein 53 (TP53), which acts as a multifunctional protein that regulates cell cycle, DNA repair, and apoptosis. We found a similar increased expression of *tp53* with those of AO-positive cells both in *rfc2* KO and *rfc5* KO zebrafish (Fig. 4A). Using Image-based Tool for Counting Nuclei (ITCN) tool of ImageJ software, we quantified the number of apoptotic cells in the brain region and identified significantly increased cell death in both KO zebrafish, compared to WT siblings (Fig. 4B). These results suggested that the microcephaly phenotype in KOs likely results from DNA damage-induced apoptosis during brain development, due to the loss-of-function of *rfc2* or *rfc5*. Alternatively, the small brain could also be induced by the reduction in neural stem cells and neural progenitors (Fig. S4). Transcription factor 4 (TCF4) regulates differentiation and migration of neural progenitors, and it is associated with Pitt–Hopkins syndrome, a severe form of ASD characterized by a range of aberrant phenotypes, including severe intellectual disability, absence of speech, delayed cognitive and motor development, and dysmorphic features (Teixeira et al., 2021). We examined the expression of *tcf7l2*, a *TCF4* homolog, in KO zebrafish at 4 dpf. In both KO animals, we observed a notable reduction in the size of *tcf7l2*-expressing areas in the forebrain and midbrain, especially optic tectum and torus semicircularis, consistent with the RNA-seq data (Fig. S4A, S4B, S4D). Additionally, we examined the expression of a neurogenic gene *her4.1*, which is important for cell proliferation, differentiation, and survival (El-Gamal et al., 2021) and crucial for neural stem cell self-renewal and neuronal differentiation (Takke et al., 1999). Expression analysis with *her4.1* indicated reduced neurogenesis in the optic tectum at 4 dpf, which aligns with the RNA-seq data (Fig. S4C and S4D).

### *rfc2* KO zebrafish have altered gene expression profiles

Based on the expression pattern of *rfc2* in zebrafish (Fig. 2A), we hypothesized that impaired RFC2 function might affect neurodevelopment and eye development. To this end, we performed RNA-seq analysis on total RNA obtained from WT and *rfc2* KO larvae harvested at 4 dpf (Table S1). As expected, *rfc2* itself was significantly reduced in *rfc2* KO zebrafish, while *rfc5* has remained unchanged (Fig. 4C). Corresponding to the cell death data (Fig. 4A and 4B), *tp53*, *mdm2*, and *cdkn1a* showed significantly augmented expression together with *clu*, which encodes clusterin protein, in *rfc2* KO zebrafish (Fig. 4C and 4D). Clusterin is known to have protective effects against apoptosis and inflammation (Palihati et al., 2024), and we previously reported that the overexpression of *clu* mRNA is induced by neuronal cell death in zebrafish (Jeong et al., 2014). To further understand the molecular network of differentially expressed genes (DEGs) from the RNA-seq analysis, we explored gene expression information in the zebrafish information network (ZFIN) database (https://zfin.org/). We established a gene expression profile against the top 313 DEGs out of 37,369 transcripts in the RNA-seq data and identified a distinct tissue-specific pattern of expression in several DEG groups, particularly related to eye and retina development (Fig. S3A). Interestingly, most retinal genes were down-regulated in *rfc2* KO zebrafish, while no significant alteration in genes involved in lens formation, such as *crystallin* genes (Fig. 4C and 4D). Among eye/retina DEG genes, several transcription factors involved in retinal neurogenesis were down-regulated: *ikzf1*, *pax6*, and *vsx* for the retinal progenitor cells, *atoh7* for the neural competence, and *pou4f2* and *isl1* for the neuronal specification, respectively. Corresponding to the RNA-seq results, we also found that the size of the lens was not significantly altered in *rfc2* KO, compared to that of WT (Fig. S3B). Moreover, in the case of genes involved in pancreas development, genes expressed in the exocrine pancreas were down-regulated (*try*, *tryl*, *try2*, *ela2*, *ela2l*, etc.), but no significant change in the endocrine pancreas-related genes (*ins*, *gcga*, *sst2*, and *dkk3b*) (Tiso et al., 2009) (Fig. 4C and 4D). Further studies will be valuable in exploring a common regulatory system for differential gene expression in different adjacent tissues within the same organ.

### Skeletal and vascular phenotypes in *rfc2* KO and *rfc5* KO zebrafish

**Fig. 5:**
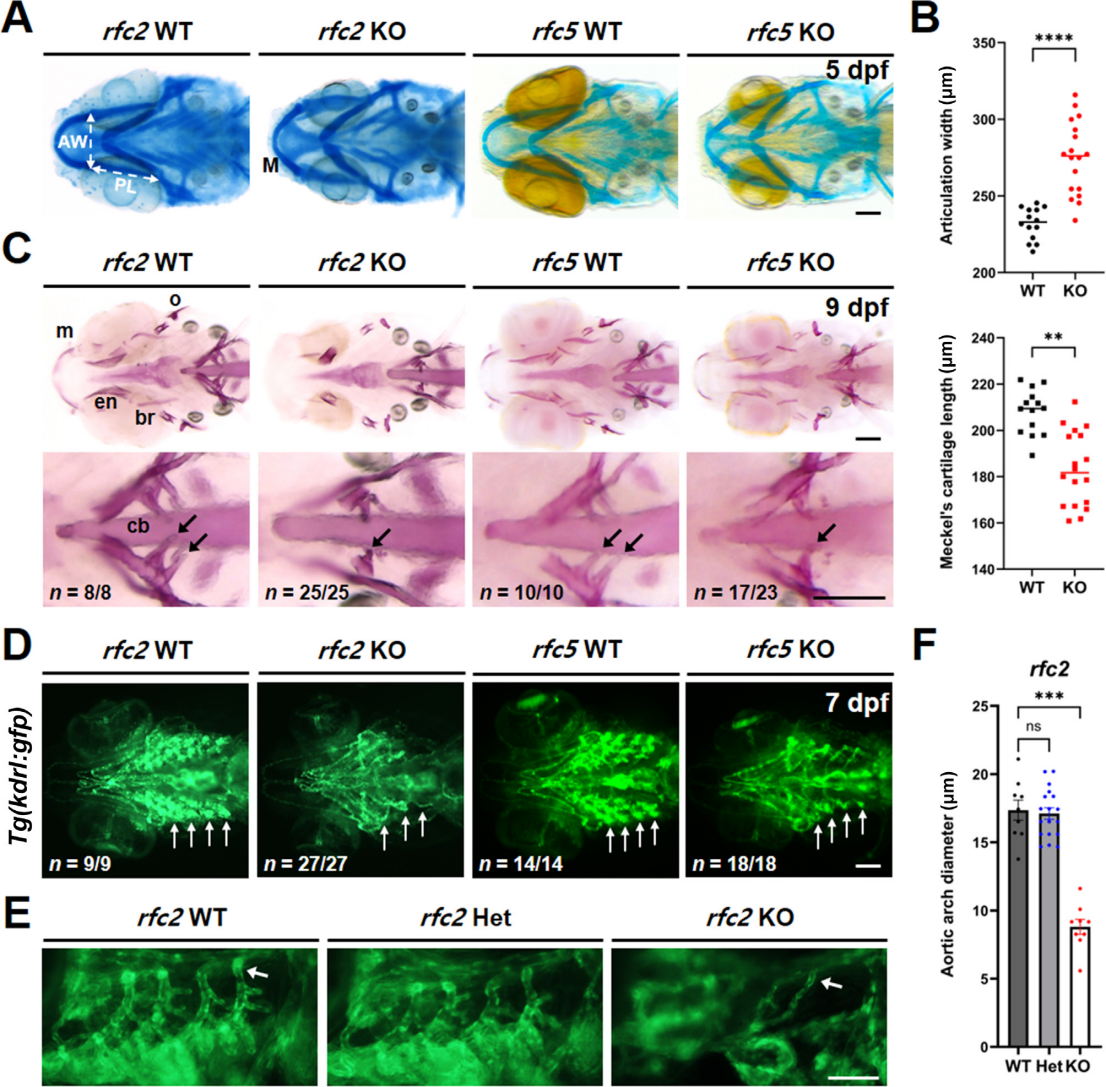
Skeletal and vascular phenotypes in *rfc2* KO and *rfc5* KO zebrafish. **A**: Defects in head cartilage formation in KO zebrafish at 5 dpf. Alcian blue staining, ventral view, anterior is to the left. **B**: Quantification of cartilage structures in *rfc2* KO zebrafish. *n* = 14 for WT and *n* = 18 for KO. **C**: Defects in bone structures in KO zebrafish. Alizarin red S staining at 9 dpf. Lower panels are magnifications of upper panels, showing defects in bone and teeth development (arrows). *n* = 8 for *rfc2* WT, *n* = 25 for *rfc2* KO, *n* = 10 for *rfc5* WT, and *n* = 23 for *rfc5* KO. Ventral view, anterior is to the left. **D**: Vascular defects in KO zebrafish crossed with *Tg(kdrl:gfp)* at 7 dpf. Aortic arches are indicated by arrows. *n* = 9 for *rfc2* WT, *n* = 27 for *rfc2* KO, *n* = 14 for *rfc5* WT, and *n* = 18 for *rfc5* KO. Ventral view, anterior is to the left. **E**: Lateral view of aortic arches. Blood vessels were narrowed and hypo-branched in *rfc2* KO, compared to WT and Het. **F**: Quantification of vessel diameter of the most posterior aortic arch (arrow in **E**) was measured using Image J software. *n* = 9 for WT, *n* = 18 for Het, and *n* = 9 for KO. Data are presented as mean ± SEM. Statistical significance was determined by the Kruskal–Wallis tests with post-hoc Dunn's multiple comparisons tests. ns, not significant, *P* > 0.05; ∗∗, *P* < 0.01; ∗∗∗, *P* < 0.001; ∗∗∗∗, *P* < 0.0001. Scale bar, 100 μm (**A**, **C**, **D**, **E**). dpf, days post fertilization; AW, articulation width; PL, Palatoquadrate length; M, Meckel's cartilage; m, maxilla; o, opercle; en, entopterygoid; br, branchiostegal ray 1; cb, ceratobranchial 5; WT, wild-type; KO, knockout; Het, heterozygous; SEM, standard error of the mean.

Facial dysmorphology is a key diagnostic feature in patients with WS involving skeletal components (Copes et al., 2016; Palmieri et al., 2019). To investigate facial skeletal dysmorphism in KO zebrafish, we examined cartilage development by alcian blue staining at 5 dpf (Fig. 5A). We found distinct abnormalities in head skeletal structures and ceratobranchial development. While there was no significant difference in the length of palatoquadrate between genotypes, the width between articulations was widened, and the length of Meckel's cartilage was shortened in KO zebrafish (Fig. 5A and 5B). We further examined bone and teeth development in 9 dpf zebrafish larvae. The alizarin red S staining indicated various defects, predominantly affecting the development of lower jaw structures such as the maxilla, opercle, entopterygoid, branchiostegal ray 1, and ceratobranchials, also known as pharyngeal jaws (Fig. 5C). Interestingly, similar defects to those seen in WS, such as microdontia and hypodontia (Ferreira et al., 2018), were observed in zebrafish teeth development on the ceratobranchial 5 of KO zebrafish, providing insights into the genetic basis of these skeletal abnormalities. Since cardiovascular problems are general in WS patients, we also examined blood vessel development in KO zebrafish. To examine the vasculature formation, we crossbred KO zebrafish with an endothelial-specific *Tg(kdrl:gfp)* transgenic line (Beis et al., 2005) to visualize vascular development in larvae at 7 dpf. Live fluorescence imaging revealed no significant differences in other blood vessels in the body trunk region, except for severe defects in the vasculature of aortic arches in KO zebrafish, compared to WT siblings (Fig. 5D–5F).

### Behavioral test in *rfc2* KO and *rfc5* KO zebrafish larvae

Given the observed anatomical and molecular-level defects in specific brain regions of *rfc2* and *rfc5* KO models, we anticipated behavioral changes in KO zebrafish. Affected regions included the pallium, which processes sensory information and regulates behavior; the thalamus, a relay center for sensory and motor signals; the hypothalamus, which manages hormone secretion and various physiological processes like feeding and circadian rhythms; the optic tectum, responsible for integrating visual information and coordinating eye movements; and the cerebellum, which ensures motor coordination and balance (Mueller, 2012; Heap et al., 2017; Kunst et al., 2019; Nagpal et al., 2019; Yanez et al., 2022). To assess the impact of these defects at larval stages, we performed circadian rhythm and dark-flash tests at 7 dpf to evaluate responsiveness and behavioral patterns of larval zebrafish to light stimulus (Ben-Moshe et al., 2014; Basnet et al., 2019; Krylov et al., 2021; Yao et al., 2023). The circadian rhythm test assessed 24-h sleep/wake behaviors under simulated natural light conditions. Locomotion activity of KO zebrafish was monitored from 14:00 on 7 dpf to 14:00 on 8 dpf. The total distance moved was measured focusing on the movement during the night of 7 and daytime of day 8. The total distance moved during each period was comparable between the WT and KO groups (Fig. S2A). Additionally, to assess reactions to light/dark transitions, larvae underwent 10 min of dark adaptation followed by 30-s light/dark intervals at 7 dpf. The measured distance moved showed no significant differences in responsiveness to light stimulation between the WT and KO groups (Fig. S2B).

### Behavioral test in heterozygous *rfc2* KO adult zebrafish

**Fig. 6:**
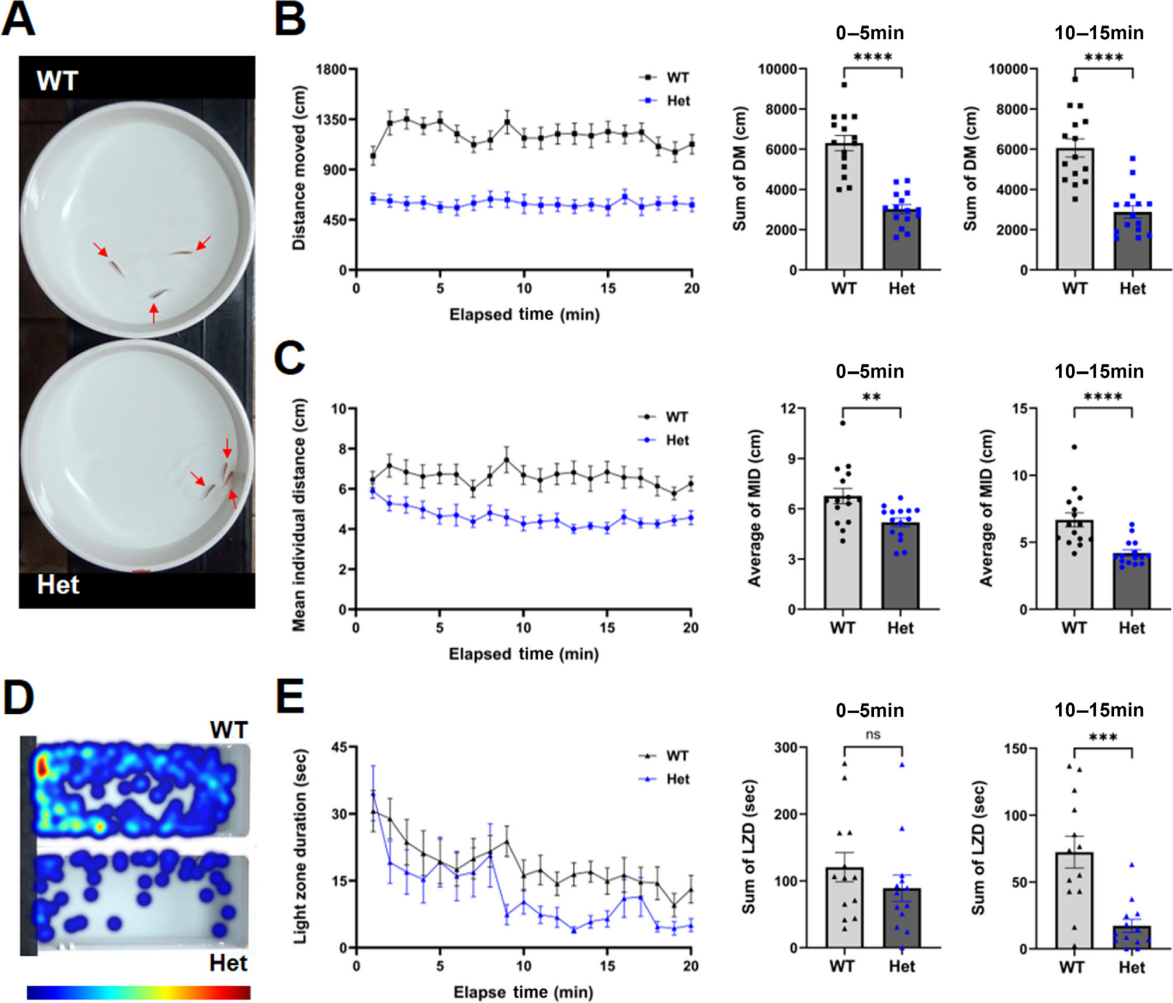
Increased social cohesion and anxiety-like behavior in Het adult *rfc2* KO zebrafish. **A**: Representative image of the shoaling bowl assay. Each arrow indicates a single zebrafish. **B**: Quantification of DM of zebrafish during shoaling bowl assay. Compared to WT siblings, *rfc2* KO Het zebrafish showed a significant reduction in their movement during the test. *n* = 5 for WT and *n* = 9 for Het fish in 15 times of tests. **C**: Quantification of MID between three subjects. Compared to WT siblings, *rfc2* KO Het tend to stay closer each other. **D**: Heatmap of scototaxis test. The red color indicates increased dwell time and blue indicates decreased. **E**: Quantification of duration time in the light zone during scototaxis test. Compared to WT siblings, *rfc2* KO Het zebrafish stayed more time in the dark zone than the light zone. *n* = 5 for WT and *n* = 9 for Het fish in total 13 times of tests. Data are presented as mean ± SEM. Statistical significance was determined by the Mann Whitney *U* test. ns, not significant, *P* > 0.05; ∗∗, *P* < 0.01; ∗∗∗, *P* < 0.001; ∗∗∗∗, *P* < 0.0001. MID, mean individual distance; Het, heterozygous; DM, distance moved; LZD, light zone duration; WT, wild-type; KO, knockout; Het, heterozygous; SEM, standard error of the mean.

Patients with WS are known for their hyper-sociability, also referred to as approachability (Doyle et al., 2004). However, they also tend to experience high anxiety (Ng-Cordell et al., 2018). Zebrafish, a highly social animal, tend to stay in close proximity and form groups through a behavior called shoaling (Choi et al., 2018). Therefore, we used the previously established shoaling bowl assay to evaluate social cohesion, social interaction, aggregation, and shoaling behavior in these zebrafish (Kim et al., 2017, 2024). Since homozygous *rfc2* KO zebrafish did not survive beyond two weeks, we performed social behavior tests using heterozygous *rfc2* KO adult zebrafish and found significant behavioral changes (Fig. 6A–6C). Compared to WT siblings, heterozygous *rfc2* KO showed reduced movement (Fig. 6B) and increased social cohesion behavior (Fig. 6A and 6C). Furthermore, behavioral impairment in heterozygous *rfc2* KO adults, especially reduced movement, can be attributed to either defects in physical motor activity or psychiatric issues, such as anxiety. To assess anxiety-like behavior in heterozygous *rfc2* KO adult zebrafish, we used the previously established scototaxis test. The light/dark paradigm is frequently employed to test anxiety-like behavior in fish and rodents, and zebrafish have been known to exhibit scototaxis (i.e., dark preference). Compared to WT siblings, heterozygous *rfc2* KO adult zebrafish traveled a shorter distance and spent less time in the light zone (Fig. 6D and 6E), indicating increased anxiety. These results suggest that *rfc2* deficiency may be involved in neurodevelopment and affect adult behaviors.

## Discussion

Previously, we identified RFC3 and RFC5 as interacting proteins to FAM50A, the deficiency of which causes an X-linked intellectual disability syndrome (Lee et al., 2020). Both proteins are components of the Replication Factor C complex, which is crucial for DNA replication and repair, cell proliferation, regulation of cell cycle checkpoints, and cell growth under stress (Kelman and Kelman, 2013). Recently, defects in the components of the RFC complex have been linked to neurodevelopmental disorders.

In this study, we identified five patients with typical 1.4 Mb–1.5 Mb WSCR microdeletions that include *RFC2* (Fig. 1). These patients showed WS phenotypes, including characteristic facial features, microcephaly or encephalopathy, intellectual disability, dental features, and cardiovascular issues. Furthermore, we identified one patient with a smaller 167.21-kb microdeletion within the WSCR, including *EIF4H*, *LAT2*, *RFC2*, and *CLIP2*, but not comprising the *ELN* gene and segregating in the family with non-syndromic intellectual disability. Also, we identified six patients with intragenic variants within *RFC2*, suggesting a potential role for *RFC2* in neurodevelopmental disorders, including ADHD, speech delay, and ASD. However, further analysis of their genomic backgrounds is essential to rule out the possible influence of other mutations.

Using zebrafish as a model, we confirmed that *rfc2* and *rfc5* mRNAs are expressed in the central nervous system (CNS), predominantly in the midbrain, MHB, hindbrain, and eyes during early development (Fig. 2). This expression pattern suggests their potential roles in CNS development. To further investigate the function of *RFC* genes in WS and typical development, we established KO models in zebrafish. Patients with WS frequently exhibit features like microcephaly or encephalopathy and reduced head and brain size (Jackowski et al., 2009), which mirrors the phenotypes observed in both *rfc2* and *rfc5* KO zebrafish (Fig. 3). These results indicate that deficiency in either gene may contribute to the microcephaly phenotype. Given that *rfc2* and *rfc5* are prominently expressed in the MHB, a region known for intense cell proliferation (Gutzman et al., 2008), we investigated whether cell death or apoptosis in this area could explain the reduced brain size in the KO models. We observed apoptosis in the same regions where *rfc2* and *rfc5* are expressed, suggesting that DNA damage-induced apoptosis during brain development might underlie the microcephaly phenotype in KOs. RNA-seq analysis further revealed the significantly augmented expression of p53 pathway markers, *tp53*, *mdm2*, and *cdkn1a*. Interestingly, many retinal genes were down-regulated in *rfc2* KO zebrafish, while no significant alteration in genes involved in lens formation (Fig. 4). Retinal ganglion cells (RGCs) are the first retinal neurons generated during development, followed by the other retinal cell types (Oliveira-Valenca et al., 2020). Transcription factors play crucial roles in the specification of RGCs from early multipotent retinal progenitor cells (*ikzf1*, *pax6*, and *vsx*) to competence stage (*atoh7*), specification (*pou4f2* and *isl1*) and terminally differentiated neurons. These findings suggest that the loss of *rfc2* and *rfc5* impairs neural cell survival and differentiation, leading to neurological damage in specific brain regions.

Facial dysmorphology is a key diagnostic feature in patients with WS. To explore this, we examined the head skeletal structures in *rfc2* and *rfc5* KO zebrafish (Fig. 5). The cartilage and bone staining in KO zebrafish showed various defects, predominantly in the development of lower jaw structures such as the maxilla, opercle, entopterygoid, branchiostegal ray 1, and pharyngeal jaws. Interestingly, similar defects to those seen in WS, such as microdontia and hypodontia (Ferreira et al., 2018), were observed in the teeth development in both *rfc2* and *rfc5* KO zebrafish, providing insights into the genetic basis of these skeletal abnormalities. Additionally, given that cardiovascular defects are another characteristic phenotype in WS patients, we also examined blood vessels in the KO zebrafish. We found severe defects in the vasculature of the aortic arches in both *rfc2* and *rfc5* KO zebrafish compared to their WT siblings.

Given the observed anatomical and molecular-level defects in specific brain regions of *rfc2* and *rfc5* KO models, we anticipated behavioral changes in these KO mutants (Figs. 6 and S2). To assess the impact of these defects at larval stages, we performed circadian rhythm and dark-flash test at 7 dpf to evaluate responsiveness and behavioral patterns of larval zebrafish to light stimulation (Ben-Moshe et al., 2014; Basnet et al., 2019; Krylov et al., 2021). In both tests (Fig. S2), the distance moved showed no significant differences between the WT and KO groups, indicating comparable responsiveness to light stimulation at the larval stage. Previously, a possible role for a small deletion between *ABHD11* and *RFC2* (∼500 kb) has been suggested in social cognition and hyper-sociability based on brain imaging and behavioral data from six subjects. (Hoeft et al., 2014). Interestingly, we found that heterozygous *rfc2* KO adult zebrafish showed an increased social cohesion behavior, similar to the hyper-sociability of WS (Fig. 6). Furthermore, the heterozygous *rfc2* KO zebrafish exhibited significant behavioral changes, especially a reduction in movement and increased anxiety-like behavior, suggesting a possible involvement of *RFC2* haploinsufficiency in neurodevelopmental disorders.

A recent study of neuropsychological evaluation in patients with atypical WSCR deletions revealed variability of the cognitive and behavioral profile of WS, depending on the genes involved. In the cases that presented isolated loss of *LIMK1*-containing region, no alterations in intellectual capacity, attention, language, memory, or executive functions were observed. However, hyper-sociability was present in most cases (Serrano-Juárez et al., 2022), including the *LIMK1*–*RFC2* region (∼200 kb) (Martindale et al., 2000). In the *Limk1*-KO mice model, however, visuospatial alterations are expressed only when both copies of the gene are missing (Osborne, 2010). It is thus believed that the alteration of additional genes is necessary for cognitive alterations. In the current study, we observed only adult behavioral changes in heterozygous *rfc2* KO zebrafish, whereas homozygous KO zebrafish showed severe craniofacial phenotypes and defects in vascular structures. Recently, bi-allelic loss-of-function variants in *RFC4* were reported, suggesting a deficiency in a gene encoding one of the small subunits of the RFC complex (RFC2–RFC5) may be involved in neurological rare disorders (Morimoto et al., 2024). Thus, heterozygous loss of *RFC2* may also be relevant when explaining cognitive defects observed in WS patients.

In summary, our study identified several patients carrying anomalies in *RFC2*, and our KO zebrafish model exhibited phenotypes that partly resemble those observed in WS. These findings suggest that the lack of *RFC2* function may contribute to certain deficits associated with WS. Moreover, KO zebrafish lines can serve as effective disease models for further characterization of the molecular mechanisms underlying WS and functional analysis of pathogenic variants. However, the specific role of the interaction between RFC proteins and FAM50A, a spliceosomal protein, in relation to neurodevelopmental disorders remains unclear. In addition to *FAM50A*, recent studies have highlighted intellectual disability and craniofacial characteristics in patients with defects in the components of spliceosome complex: *EFTUD2* mutations cause Guion-Almeidatype mandibulofacial dysostosis; *EIF4A3* variants cause Richieri–Costa–Pereira syndrome; *THOC2* variants result in an intellectual disability and abnormal gait (Lee et al., 2020). Given that RFC proteins can interact with the spliceosome through FAM50A, and spliceosome defects are known causes of genetic syndromes, investigating this interaction could be a promising future research avenue. This underscores the need for further studies to fully elucidate these relationships within the context of neurodevelopmental disorders.

## Materials and methods

### Ethical approval and consent

The study adhered to the tenets of the Declaration of Helsinki, and Institutional Review Board (IRB) approval was obtained from Pusan National University Yangsan Hospital (IRB No. 05-2023-138), Chungnam National University Sejong Hospital (IRB No. 2022-04-008), and Baylor College of Medicine (H-36612). Informed consent was obtained for patients 1–5 for publication and genetic analyses. A waiver of informed consent has been obtained for subjects 7–12 because the analysis and publication of human subject data included in this study have been de-identified and present minimal risks to the study subjects.

### Genetic analysis

A blood sample was collected for DNA extraction, and informed consent was obtained from patients 1–5 before the blood was drawn. A chromosomal microarray test (CMA) was used for the detection of microdeletions in the patients. Patients were diagnosed with WS on the CMA test (CytoScan Dx Assays), resulting in microdeletions on chromosome 7q11.23 (reference genome GRCh37/hg19). Subjects 7–10 were referred to clinical testing because of suspicion of an underlying genetic disorder. Their DNA was analyzed on a clinical CMA as previously described (Yuan et al., 2020). Subjects 11 and 12 were referred to clinical whole-exome sequencing because of suspicion of an underlying genetic disorder (Yang et al., 2014).

### Zebrafish maintenance

Zebrafish were reared in our animal facility under standard conditions at 28.5°C with a 14-h light/10-h dark cycle. Embryos were obtained and reared in egg water at 28.5°C incubator. Developmental stages of the organogenesis period are represented as hours post fertilization (hpf). We obtained wild-type and transgenic zebrafish, *Tg(huc:egfp)* and *Tg(kdrl:gfp)*, from the Zebrafish Center for Disease Modeling (ZCDM), South Korea. All zebrafish experiments were conducted in accordance with the approved guidelines and regulations of the Institutional Animal Care and Use Committee (IACUC) at the Animal Ethics Committee of Chungnam National University (202404A-CNU-077).

### Expression analysis with whole-mount in situ hybridization

Whole-mount in situ hybridization was performed using anti-sense digoxigenin-labeled RNA probes (Thisse et al., 2004). Staged zebrafish embryos were fixed overnight in 4% paraformaldehyde (PFA) (Sigma-Aldrich, P6148) in phosphate-buffered saline (PBS, pH 7.4) at 4°C Fixed embryos were washed with DEPC (Diethylpyrocarbonate, Sigma-Aldrich, D5758)-PBST (PBS with 0.1% Tween 20) and then dehydrated with stepwise 25/50/75/100% methanol (Honeywell, 34966). Dehydrated embryos were stored in 100% methanol at −20°C until usage. Embryos were rehydrated in PBST and permeabilized by digestion with 10 μg/mL proteinase K (Roche, 3115828001). Permeabilized embryos were hybridized overnight with 50 ng digoxigenin-labeled probes at 70°C. The next day, hybridized embryos were washed with a preheated mixture of 50% hybridization solution and 50% saline sodium citrate (SSC) containing 0.1% Tween-20 at 70°C, followed by incubation with 1/4000 Anti-digoxigenin AP-conjugate (Roche, 11093274910). Finally, embryos were washed 6 times with PBST and incubated in NTMT staining buffer (0.1 M NaCl, 0.1 M Tris-Cl, 50 mM MgCl_2_, 0.1% Tween-20) at room temperature. Incubated embryos were stained with staining solution (4.5 μL NBT + 3.5 μL BCIP/1 mL staining buffer) in dark conditions until sufficient staining appeared. Embryos were mounted in 90% glycerol for imaging and imaged under M205 FA microscope (Leica Microsystems, Germany).

### Generation of *rfc2* and *rfc5* KO zebrafish using CRISPR-Cas9

To identify the *RFC2* and *RFC5* zebrafish ortholog, we performed reciprocal BLAST of human RFC2 protein (NCBI: NP_852136.1) and human RFC5 protein (NCBI: NP_031396.1) against the zebrafish genome and identified Rfc2 with 90% similarity and 85% identity (NCBI: NP_001013344.2) and Rfc5 with 91% similarity and 82% identity (NCBI: NP_001003862.1).

To understand the in vivo role of Rfc2 and Rfc5, we used the CRISPR-Cas9 system to generate *rfc2* and *rfc5* KO zebrafish. Oligonucleotides to synthesize sgRNAs targeting exon 1 of *rfc2* (5′-AGCTGACGGACCGCCTAAAAAGG-3′) and exon 3 of *rfc5* (5′-CCTCATCTGCTCTTCTATGGACC-3′) were designed by CRISPRscan (https://www.Crisprscan.org/). In vitro transcription of sgRNA was conducted using MAXIscript™ T7 Transcription Kit (Invitrogen, AM1314), and synthesized RNA was purified with 5 M ammonium acetate. Cas9 expression vector (pT3TS-nCas9n) was linearized with *Xba*I (NEB, R0145) and purified with 0.3 M sodium acetate. Cas9 mRNA was transcribed with mMESSAGE mMACHINE™ T3 Transcription Kit (Invitrogen, AM1348), poly(A) tailed with Poly(A) Tailing Kit (Invitrogen, AM1350) and then purified by lithium chloride precipitation. One-cell stage zebrafish embryos were injected with 300 ng/μL Cas9 mRNA and 100 ng/μL sgRNA. To check the efficiency of Cas9 mRNA and sgRNA, genomic DNA of injected embryos was isolated using the HotSHOT method, amplified by PCR using the following primer pairs, *rfc2*-injected with 5′-TGTTTTGTTC CTGTCGTGGC-3′ (forward) and 5′-TAGCCACATGAATC CCGTCC -3′ (reverse), *rfc5*-injected with 5′-AGTGAAGACCGACTGCCTC-3′ (forward) and 5′-CAGGACCATGGAGTTGA ACT-3′ (reverse), respectively. Amplified fragments were re-annealed and digested with T7 endonuclease I (NEB, M0302L) to identify somatic mutations. Identified founder F0 zebrafish were crossed with WT zebrafish, and germline transmission resulted in the propagation of *rfc2* ^ck179a^ KO line with 20-bp insertion (22-bp insertion with 2-bp deletion), *rfc2* ^ck179b^ KO line with 73-bp deletion and *rfc5* ^ck171a^ KO line with 8-bp deletion, *rfc5* ^ck171b^ KO line with 10-bp insertion (11-bp insertion with 1-bp deletion).

### Acridine orange staining for cell death

Acridine orange staining was performed to detect apoptotic cells in live zebrafish larvae (Tucker and Lardelli, 2007). Embryos were reared in egg water from fertilization to 10 hpf, and in 0.2 mM PTU (N-Phenylthiourea, Sigma-Aldrich, P7629) to prevent pigmentation, which can disturb clear imaging. PTU-treated embryos were collected in 1.5 mL microcentrifuge tube and washed 5 times with egg water for 5 min each. Washed embryos were stained for 30 min in dark condition with 10 μg/mL acridine orange (Sigma-Aldrich, A6014) solution, followed by 5 times washing with egg water for 5 min each. Washed embryos were anesthetized and mounted on 3% methylcellulose for imaging under CELENA® S digital microscope (Logos Biosystems, Korea).

### Cartilage and bone staining

Alcian blue staining was conducted to stain the cartilage of zebrafish larvae (Kim et al., 2000). Embryos at 5 dpf were fixed overnight in 4% PFA at 4°C. Fixed embryos were bleached with bleaching solution (3% H_2_O_2_, 0.5% KOH) for 1 h, washed 3 times with PBST for 5 min each and dehydrated with methanol until usage. Stored embryos were re-hydrated with PBST and washed with 100%, 50% ethanol for 5 min each, followed by staining for 3 h with 0.02% alcian blue (Sigma-Aldrich, A5268) solution. Stained embryos were washed 3 times with 100% ethanol for 5 min each and then bleached to remove non-specific staining, followed by 3 times washing with PBST. Embryos were mounted in 90% glycerol for imaging and imaged under M205 FA microscope (Leica Microsystems, Germany). The calcified bone structure of larval zebrafish was stained with alizarin red S. Zebrafish larvae at 9 dpf were fixed briefly in 4% PFA at room temperature and washed 5 times with PBST for 5 min each. Fixed embryos were bleached with bleaching solution (3% H_2_O_2_, 0.5% KOH) and washed 5 times with PBST for 5 min each, followed by staining with alizarin red S solution for 30 min. Stained embryos were de-stained twice with 0.5% KOH for 5 min each and then mounted in 90% glycerol for imaging. Images were taken under M205 FA microscope (Leica Microsystems, Germany).

### Circadian rhythm locomotion activity in larval zebrafish

Circadian rhythm locomotion activity of larval zebrafish was assessed by measuring distance moved during day-time and night-time (Rihel et al., 2010). Zebrafish larvae were raised at 28.5°C incubator with a 14-h light/10-h dark cycle until 7 dpf and placed in each well of a 48-well plate (spl, 30048) containing 1 mL of egg water. Plates were placed in the observation chamber of DanioVision (Noldus, Netherlands) for video recording. Locomotion activity was monitored for 24 h (from 2 p.m. on Day 7 to 2 p.m. on Day 8) in the same environment in which the animals were raised, and all the larvae were genotyped after the experiment. The day and night locomotion activities were analyzed every 30 min by calculating the total distance moved using EthoVision XT 17 software (Noldus, Netherlands).

### Dark-flash response test in larval zebrafish

Larval zebrafish have the tendency to decrease their locomotion in response to a sudden change in the illumination of their environment. A reduction of locomotor activity is normally observed during a period of sudden flash, referred to as a “dark-flash” response (Burgess and Granato, 2007). Briefly, larvae at 7 dpf were placed in 48-well plate inside the DanioVision and habituated to the dark condition for 10 min. After dark adaptation, larvae were exposed to five alternating flashes of light and darkness for 30 s each. The distance moved by larvae was analyzed every 10 s using EthoVision XT 17 software (Noldus, Netherlands).

### Shoaling bowl assay in adult zebrafish

The shoaling bowl assay (Kim et al., 2017, 2024) was conducted in adult zebrafish using 7-month-old male zebrafish. Three adult animals of the same genotype were placed in a round, flat bottom, white bowl (upper diameter, 33 cm; bottom diameter, 22 cm; height, 11 cm; water volume, 2 L), and top-view video was acquired for 20 min with a video camera (Sony, HDR-CX190). The distance moved and mean individual distance were measured in video frames taken every 1 min using EthoVision XT 17 software (Noldus, Netherlands).

### Scototaxis test in adult zebrafish

The scototaxis (dark/light preference) test was modified and improved upon from previous studies (Maximino et al., 2010; Choi et al., 2018) using 9-month-old male zebrafish. Zebrafish were placed in a tank (47 cm × 16 cm × 12 cm, and water volume 4.5 L) divided in half into light zone and dark zone. The light zone was covered with non-transparent white paper, and the dark zone was covered with black, upper with the black plastic lid. A total of 20 min of top-view video was taken using a video camera (Sony, HDR-CX190). The light zone duration time and distance moved in the light zone were measured in every 1 min using EthoVision XT 17 software (Noldus, The Netherlands).

### Statistical analysis

All statistical analyses were performed using Prism 10 (GraphPad, USA). Data was presented as mean ± standard error of the mean (SEM). Statistical significance was determined by the unpaired two-tailed *t*-tests with Welch's correction, the Mann Whitney *U* test, or the Kruskal-Wallis test with post-hoc Dunn's multiple comparisons tests. ns, not significant, *P* > 0.05; ∗, *P* < 0.05; ∗∗, *P* < 0.01; ∗∗∗, *P* < 0.001; ∗∗∗∗, *P* < 0.0001.

### RNA-seq and gene expression analysis

A cDNA library was independently prepared with total RNA for each sample by Illumina TruSeq Stranded Total RNA Library Prep Gold Kit (Illumina, Inc., San Diego, CA, USA, #20020599). Cleaved RNA fragments are copied into first-strand cDNA using SuperScript II reverse transcriptase (Invitrogen, #18064014), followed by second-strand cDNA synthesis. Indexed libraries were then submitted to an Illumina NovaSeq (Illumina, Inc., San Diego, CA, USA), and the paired-end (2 × 150 bp) sequencing was performed by Macrogen Incorporated. Paired-end sequencing reads were generated on the Illumina sequencing NovaSeq platform. Cleaned reads were aligned to the *Danio rerio* (GRCz11) using HISAT v2.1.0 (Kim et al., 2015). The reference genome sequence and gene annotation data were downloaded from the NCBI Genome assembly and NCBI RefSeq database, respectively. After alignment, the transcripts were assembled and quantified using StringTie v2.1.3b (Pertea et al., 2015, 2016). Gene-level and Transcript-level quantification were calculated as raw read count, fragments per kilobase of transcript per million mapped reads (FPKM), and transcripts per million (TPM). Statistical analyses of differential gene expression were performed by edgeR exactTest (Robinson et al., 2010). Principal component analysis (PCA) and multidimensional scaling (MDS) plots were generated to confirm the similarity of expression between samples. The statistical significance of differential expression gene was determined using edgeR exactTest. Fold change and *P*-value were extracted from the result of exactTest. All *P*-values are adjusted by the Benjamini-Hochberg algorithm to control the false discovery rate (FDR). The significantly differentially expressed genes were filtered by |fold change| ≥ 2 and raw *P*-value < 0.05. Gene-enrichment and functional annotation analysis for significant genes were carried out using gProfiler (Raudvere et al., 2019) against the Gene Ontology (GO) database and using the in-house KEGG Viewer script against KEGG pathway database (http://www.genome.jp/kegg/pathway.html). All data analysis and visualization of differentially expressed genes was conducted using R 4.2.2 (www.r-project.org).

## Data availability

All data are available from the corresponding authors upon reasonable request. Transcriptomic data was deposited in the NCBI database under BioProject accession number PRJNA1165428.

## CRediT authorship contribution statement

**Ji-Won Park**: Writing - Original draft, Investigation, Formal analysis. **Tae-Ik Choi**, **Tae-Yoon Kim**, **Yu-Ri Lee**, **Dilan Wellalage Don**: Investigation. **Jaya K. George-Abraham**, **Laurie A. Robak**, **Cristina C. Trandafir**, **Pengfei Liu**: Formal analysis. **Jill A. Rosenfeld**: Investigation, Formal analysis. **Tae Hyeong Kim** and **Florence Petit**: Formal analysis. **Yoo-Mi Kim** and **Chong Kun Cheon**: Writing - Review & Editing, Investigation, Funding acquisition. **Yoonsung Lee**: Writing - Review & Editing, Funding acquisition. **Cheol-Hee Kim**: Writing - Review & Editing, Funding acquisition, Conceptualization.

## Conflict of interest

The Department of Molecular and Human Genetics at Baylor College of Medicine receives revenue from clinical genetic testing completed at Baylor Genetics.

## Acknowledgments

This work was supported by the 10.13039/501100003725National Research Foundation of Korea grant funded by the Korean government (MIST) (2020R1A5A8017671, 2022R1A2C100677813, and RS-2024-00349650). C.K.C. was supported by a 2023 research grant from Pusan National University Yangsan Hospital. Y.M.K. was supported by the 10.13039/501100003653Korea National Institute of Health research project (No. 2022ER050800). This study makes use of data generated by the DECIPHER community (Firth et al., 2009). A full list of centers who contributed to the generation of the data is available from https://deciphergenomics.org/about/stats and via email from contact@deciphergenomics.org. DECIPHER is hosted by EMBL-EBI and funding for the DECIPHER project was provided by the Wellcome Trust (grant number WT223718/Z/21/Z).
