# Supplementary material for: RFC2 may contribute to the pathogenicity of Williams syndrome revealed in a zebrafish model
Source: J Genet Genomics. 2024 Dec;51(12):1389–403. doi: 10.1016/j.jgg.2024.09.016 (PMC11624490; doi:10.1016/j.jgg.2024.09.016)
Supplement: Multimedia component 1 [file mmc1.docx]

***RFC2* may contribute to the pathogenicity of Williams syndrome revealed in a zebrafish model**

Ji-Won Park ^a^, Tae-Ik Choi ^a^, Tae-Yoon Kim ^a^, Yu-Ri Lee ^a^, Dilan Wellalage Don ^a^, Jaya K George-Abraham ^b^, Laurie A Robak ^c^, Cristina C Trandafir ^c^, Pengfei Liu ^c,d^, Jill A Rosenfeld ^c,d^, Tae Hyeong Kim ^e^, Florence Petit ^f^, Yoo-Mi Kim ^g,*^, Chong Kun Cheon ^h,i,*^, Yoonsung Lee ^j*^, Cheol-Hee Kim ^a,*^

^a^ Department of Biology, Chungnam National University, Daejeon, Republic of Korea

^b^ Department of Pediatrics, The University of Texas at Austin Dell Medical School, Austin, TX, USA

^c^ Department of Molecular and Human Genetics, Baylor College of Medicine, Houston, TX, USA

^d^ Baylor Genetics Laboratories, Houston, TX, USA

^e^ Department of Pediatrics, Kyung Hee University Hospital at Gangdong, Seoul, Republic of Korea

^f^ Univ. Lille, CHU Lille, Clinique de génétique Guy Fontaine, F-59000 Lille, France

^g^ Department of Pediatrics, Chungnam National University Sejong Hospital, Sejong, Republic of Korea

^h^ Department of Pediatrics, Pusan National University Children’s Hospital, Yangsan, Republic of Korea

^i^ Research Institute for Convergence of Biomedical Science and Technology, Pusan National University Yangsan Hospital, Yangsan, Republic of Korea

^j^ Clinical Research Institute, Kyung Hee University Hospital at Gangdong, School of Medicine, Kyung Hee University, Seoul, Republic of Korea

^*^ Corresponding authors.

E-mail addresses: ym.kim@cnu.ac.kr (Y.-M. Kim), chongkun@pusan.ac.kr (C.K. Cheon), ylee3699@khu.ac.kr (Y. Lee), zebrakim@cnu.ac.kr (C.-H. Kim).


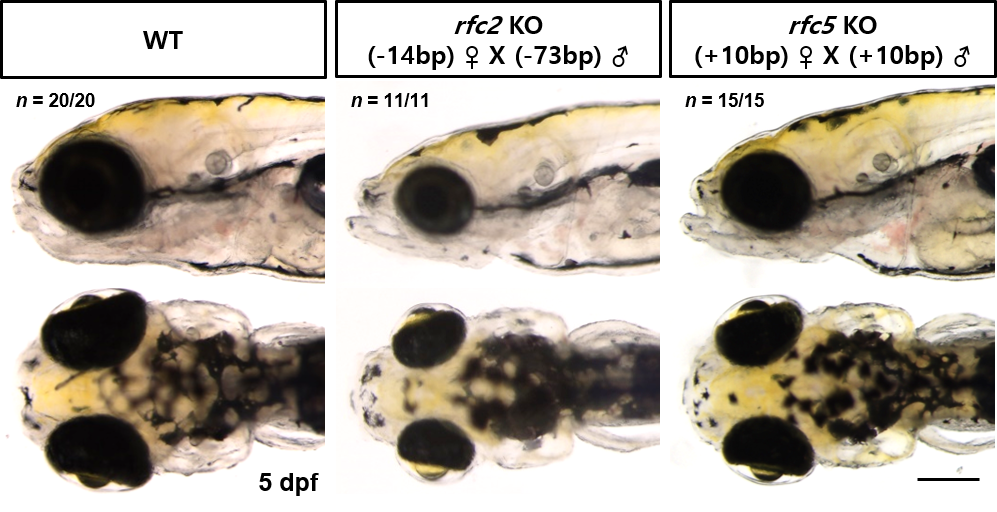
**Fig. S1.** Genetic complementary experiment by crossing different lines of *rfc2* KO and *rfc5* KO. The early morphological phenotypes of both *rfc* KOs, such as small head and small eyes, were identical to KOs of other alleles at 5 dpf. *n* = 20 for WT, *n* = 11 for *rfc2* KO, and *n* = 15 for *rfc5* KO. Scale bar, 200um.


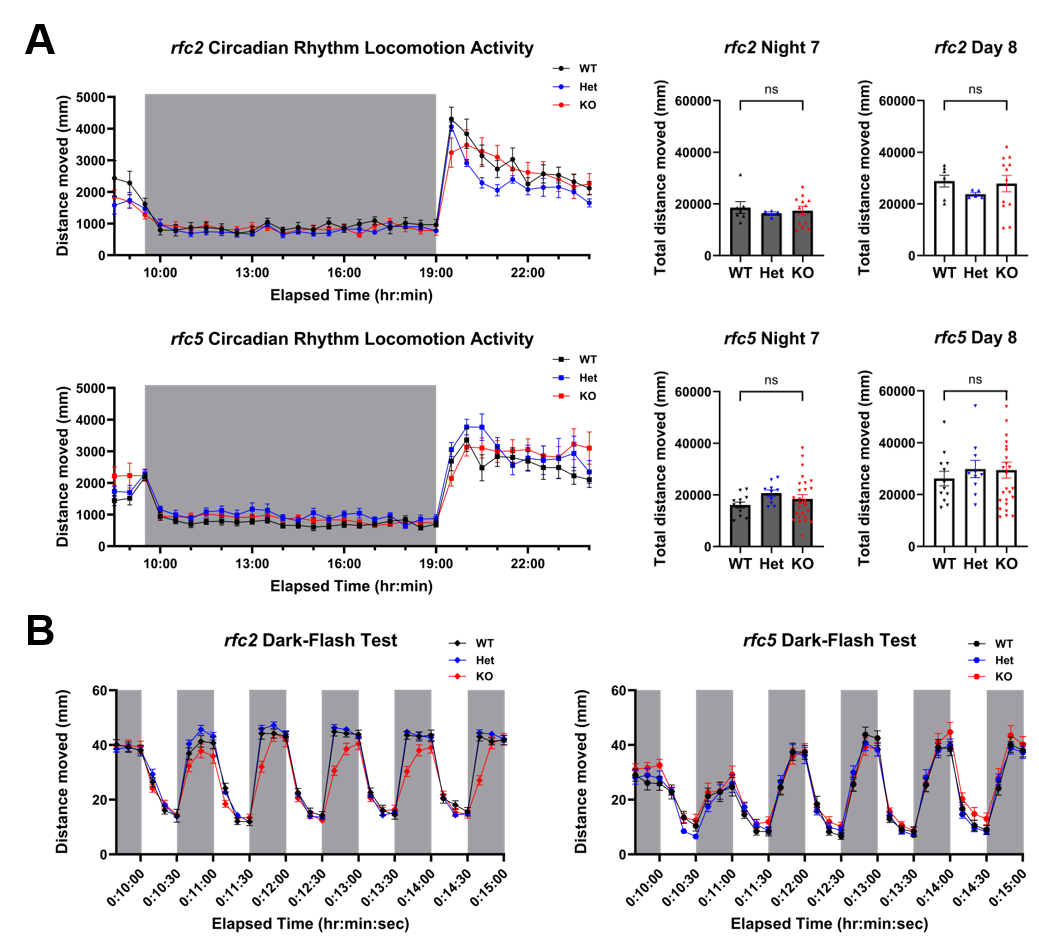
**Fig. S2.** Behavioral tests in *rfc2* KO and *rfc5* KO zebrafish larvae. **A:** Circadian rhythm test in *rfc2* KO and *rfc5* KO zebrafish at 7-8 dpf. Locomotion activity of zebrafish larvae was quantified with the distance moved during night period and day period. Both *rfc2* KO and *rfc5* KO zebrafish exhibited relatively normal circadian rhythm behavior, compared to WT and Het siblings. *n* = 7 for *rfc2* WT, *n* = 5 for *rfc2* Het, *n* = 12 for *rfc2* KO, *n* = 12 for *rfc5* WT, *n* = 10 for *rfc5* Het, and *n* = 26 for *rfc5* KO. Dark-boxes indicate night 7 period. **B:** Dark-Flash response test at 7 dpf. Startle response of zebrafish larvae was quantified with the distance moved of locomotion. *n* = 56 for *rfc2* WT, *n* = 100 for *rfc2* Het, *n* = 156 for *rfc2* KO, *n* = 46 for *rfc5* WT, *n* = 74 for *rfc5* Het, and *n* = 156 for *rfc5* KO. Data was presented as mean ± standard error of the mean (S.E.M.). Statistical significance was determined by the Kruskal–Wallis tests with post-hoc Dunn’s multiple comparisons tests. ns, not significant, *P* > 0.05.

**
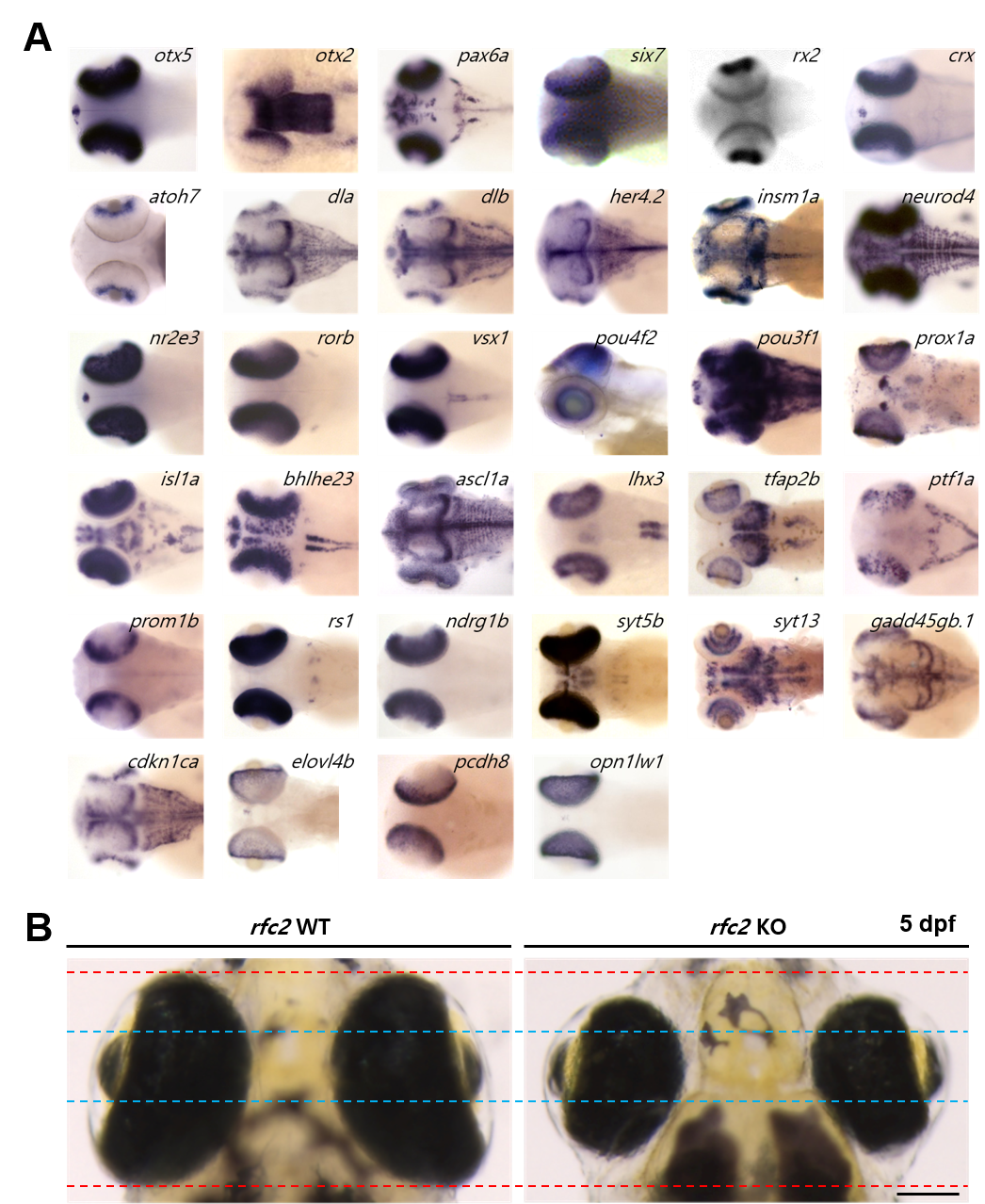
Fig. S3.** Expression profile of differentially expressed genes (DEGs) from the RNA-seq analysis. **A:** Representative images of genes involved in eye development, and retinal differentiation and function. Tissue-specific expression pattern of the top 313 DEGs out of 37,369 transcripts in the RNA-seq data was profiled after search of gene expression information in the zebrafish information network (ZFIN) database (https://zfin.org/). **B:** Comparison of eye size between WT and *rfc2* KO zebrafish at 5 dpf. Lens development was relatively normal (indicated by blue line), while eye/retina (red line) and brain size were severely reduced in *rfc2* KO zebrafish, compared to WT. *n* = 10 for WT and *n* = 11 for KO. Top view. Scale bar, 100 μm.

**
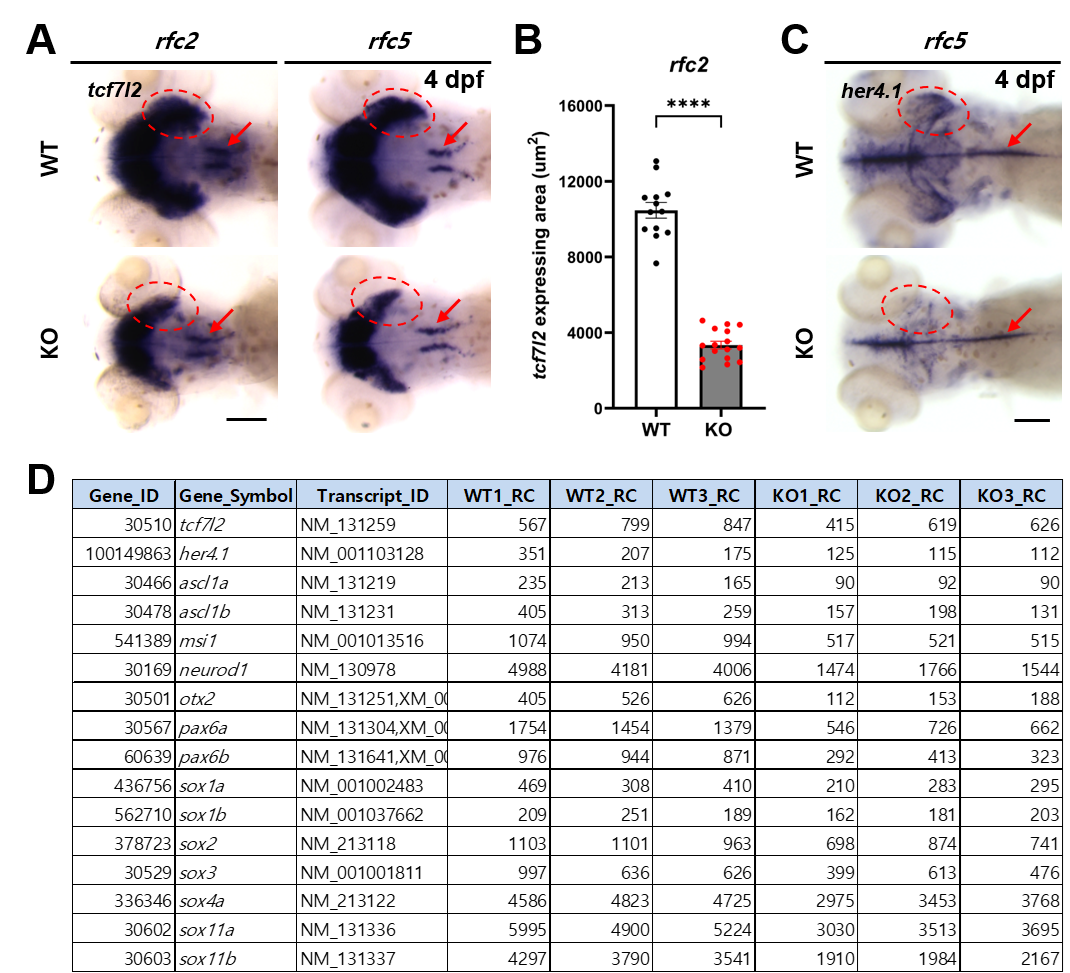
Fig. S4.** Expression analysis of genes involved in neural progenitor formation and neurogenesis. **A:** Reduced expression of neural progenitor marker *tcf7l2* in KO zebrafish at 4 dpf, especially in the torus semicircularis (TS) region (marked with circle). Its expression is less affected in hindbrain (arrows). *n* = 13 for *rfc2* WT, *n* = 16 for *rfc2* KO; *n* = 25 for *rfc5* WT, and *n* = 17 for *rfc5* KO. Dorsal view, anterior is to the left. **B:** Quantification of regional *tcf7l2* expression in *rfc2* KO zebrafish at 4 dpf. The TS area of *tcf7l2* expression in (A) was measured using image J software. *n* = 13 for WT and *n* = 16 for KO. **C:** Reduced expression of *her4.1*, a neurogenesis marker, in *rfc5* KO at 4 dpf. *n* = 10 for WT and *n* = 18 for KO. Dorsal view, anterior is to the left. **D:** Raw read count (RC) data of the RNA-seq analysis, showing differential expression of neural progenitor markers, including *tcf7l2* and *her4.1*, in *rfc2* KO zebrafish. Data are presented as mean ± standard error of the mean (S.E.M.). Statistical significance was determined by the Mann-Whitney U test. ****, *P* < 0.0001. Scale bars, 100 μm.
